# Near-Infrared Light-Triggered Generation of Reactive Oxygen Species and Induction of Local Hyperthermia from Indocyanine Green Encapsulated Mesoporous Silica-Coated Graphene Oxide for Colorectal Cancer Therapy

**DOI:** 10.3390/antiox11010174

**Published:** 2022-01-17

**Authors:** Hyung Woo Choi, Jae Hyun Lim, Chan Woo Kim, Eunmi Lee, Jin-Moo Kim, Kiyuk Chang, Bong Geun Chung

**Affiliations:** 1 Department of Mechanical Engineering, Sogang University, Baekbeom-ro 35, Mapo-gu, Seoul 04107, Korea; gmpwoo@hanmail.net; 2 Department of Biomedical Engineering, Sogang University, Baekbeom-ro 35, Mapo-gu, Seoul 04107, Korea; boy6635@naver.com; 3 Division of Cardiology, Department of Internal Medicine, Seoul St. Mary’s Hospital, College of Medicine, The Catholic University of Korea, Banpo-daero 222, Seocho-gu, Seoul 06591, Korea; cw.kim@catholic.ac.kr (C.W.K.); dmsaltks@naver.com (E.L.); amyeonsohon@nate.com (J.-M.K.); 4 Catholic Research Institute for Intractable Cardiovascular Disease, College of Medicine, The Catholic University of Korea, Banpo-daero 222, Seocho-gu, Seoul 06591, Korea

**Keywords:** mesoporous silica, reduced graphene oxide, indocyanine green, tumor targeting, photothermal and photodynamic therapy

## Abstract

Near-infrared (NIR) light-mediated photothermal therapy (PTT) and photodynamic therapy (PDT) have widely been used for cancer treatment applications. However, a number of limitations (e.g., low NIR absorption capacity of photothermal agents, insufficient loading efficiency of photosensitive molecules) have hindered the widespread use of NIR-mediated cancer therapy. Therefore, we developed a mesoporous silica-coated reduced graphene oxide (rGO) nanocomposite that could provide a high encapsulation rate of indocyanine green (ICG) and enhance PTT/PDT efficiency in vitro and in vivo. The ICG-encapsulated nanocomposite not only enhances the photothermal effect but also generates a large number of tumor toxic reactive oxygen species (ROS). By conjugation of polyethylene glycol (PEG) with folic acid (FA) as a tumor targeting moiety, we confirmed that ICG-encapsulated mesoporous silica (MS)-coated rGO nanocomposite (ICG@MS-rGO-FA) exhibited high colloidal stability and intracellular uptake in folate receptor-expressing CT-26 colorectal cancer cells. Upon NIR laser irradiation, this ICG@MS-rGO-FA nanocomposite induced the apoptosis of only CT-26 cells via enhanced PTT and PDT effects without any damage to normal cells. Furthermore, the ICG@MS-rGO-FA nanocomposite revealed satisfactory tumor targeting and biocompatibility in CT-26 tumor-bearing mice, thereby enhancing the therapeutic effects of PTT and PDT in vivo. Therefore, this tumor-targeted ICG@MS-rGO-FA nanocomposite shows a great potential for phototherapy applications.

## 1. Introduction

Over the past few decades, photodynamic therapy (PDT), a form of phototherapy, has been frequently used for cancer treatment [1,2,3]. This approach involves non-toxic photosensitizers (Ps) and localized oxygen to produce cytotoxic reactive oxygen species (ROS) under visible light irradiation (500–600 nm), causing apoptosis and necrosis of cancer cells [4,5]. Compared to conventional modalities (e.g., surgery, radiotherapy, and chemotherapy), PDT exhibits good cancer treatment efficacy because of its minimal invasiveness, excellent reproducibility, protection of vital organs, and low cytotoxicity to normal cells [6,7]. However, the low selectivity, fast decomposition, limited penetration of visible light, and hydrophobic properties of PS cannot completely eliminate cancer cells, leading to the high recurrence rate [8,9,10]. Recently, combination therapy (e.g., PDT with NIR light-mediated photothermal therapy (PTT)) is emerging as a powerful tool to develop the therapeutic efficiency and is widely applied to various cancer treatments [11,12,13,14]. The primary advantages of PTT are that various photothermal agents (e.g., gold, copper sulfide, reduced graphene oxide, and molybdenum disulfide) can effectively inhibit tumor growth and induce the apoptosis of cancer cells by local hyperthermia under NIR light irradiation (700–1000 nm) [15,16,17]. However, this combined PTT/PDT requires two different light sources (e.g., NIR and visible light), which causes systemic complexity and hinders clinical applications due to the precise alignment of two wavelength lights and the elongated treatment time [18,19,20]. Therefore, to optimize the synergistic therapeutic effect, the simultaneous activation of PTT and PDT via single laser light irradiation is necessary.

Indocyanine green (ICG), which is approved by the U.S. Food and Drug Administration (FDA), has widely been used for NIR-mediated PTT/PDT and imaging applications via single wavelength light irradiation [21,22,23]. Despite its great potential, an ICG has several intrinsic drawbacks, such as structural instability, short plasma half-life, and lack of targeting ability [24,25]. To overcome these limitations, a number of studies have focused on encapsulation or immobilization of ICG on nanoparticle platforms, leading to enhanced photochemical stability, circulation time, and therapeutic efficacy [26,27,28]. Sharker et al. reported pH- and NIR-sensitive ICG-polymer-rGO hybrid nanocomposites formed via electrostatic interactions for PTT and fluorescence imaging applications [29]. Additionally, Mazza et al. introduced ICG-encapsulated liposomes by hydrophobic interactions for tracking human mesenchymal stem cells [30]. These previous reports have focused on the encapsulation of ICG molecules into nanocomposites as a therapeutic or imaging agent. However, most nanomaterials exhibited poor encapsulation efficiency of ICG, as well as inadvertent release of phototherapeutic agents (ICG), resulting in low NIR absorption and insufficient production of cytotoxic agents, such as ROS, thereby resulting in reduced therapeutic efficacy.

Carbon-based nanomaterials (e.g., carbon nanotubes (CNTs), quantum dots (QDs), nanodiamonds (NDs), and graphene oxide (GO)) have widely been exploited for various biomedical applications, including biosensors, drug delivery, tissue engineering, imaging, and cancer therapy [31,32,33,34]. Among these nanomaterials, a two-dimensional (2D) GO has gained tremendous attention as a drug carrier and an NIR-mediated photothermal agent for cancer treatment due to its excellent electronic, physicochemical, and optical properties [35,36]. The large surface areas and π electrons in a GO-based nanomaterial enable the delivery of therapeutic agents (e.g., anticancer drug, genetic material) to provide effective cancer therapy [37,38,39,40]. Although GO-based nanomaterials may offer a solution to the aforementioned limitations (e.g., easy denaturation, poor solubility, high toxicity to normal cells, and low specificity of tumor sites), they still need to be improved for complete eradication of malignant tumors [41,42,43].

In this study, we developed ICG-encapsulated mesoporous silica-coated rGO nanocomposites with enhanced PTT and PDT dual therapeutic effects against colorectal cancer (CRC) cells. To increase the encapsulation amount of ICG molecules, a mesoporous silica (MS) was covered to form uniform pore structures on rGO nanosheets. Upon NIR laser irradiation, MS-rGO nanocomposites show the inherent PTT effect, generate large amounts of ROS, and increase the temperature via encapsulated ICG in MS-rGO nanocomposites. Moreover, the incorporation of folic acid (FA)-conjugated polyethylene glycol (PEG) onto ICG@MS-rGO nanocomposites could improve their colloidal stability and selective targeting of folate receptor-positive CRC cells, thereby improving their tumor targeting and therapeutic effects of PTT and PDT in vitro and in vivo. Therefore, this ICG@MS-rGO-FA nanocomposite could be a potentially powerful nanocarrier to enhance synergistic therapeutic efficacy.

## 2. Materials and Methods

### 2.1. Materials

GO solution (1 mg/mL) was purchased from Graphene Square, Inc. (Suwon, Korea). Hydrazine monohydrate (N_2_H_4_·H_2_O), cetyltrimethylammonium bromide (CTAB), triethanolamine (TEA), 3-aminopropyltriethoxysilane (APTES), 1-ethyl-3-(3-dimethylaminopropyl) carbodiimide (EDC), *N*-hydroxysuccinimide (NHS), and tetraethyl orthosilicate (TEOS) were obtained from Sigma-Aldrich (St. Louis, MO, USA). FA-conjugated PEG acid (MW: 5 kDa, FA-PEG-COOH) and methoxy PEG acid (MW: 5 kDa, mPEG-COOH) were purchased by a Nanocs, Inc. (New York, NY, USA). ICG and 1,3-diphenylisobenzofuran (DPBF) were purchased from Tokyo Chemical Industry Co., Ltd. (Tokyo, Japan). CT-26 CRC cells and NIH-3T3 fibroblast cells were obtained from Korea Cell Line Bank (Seoul, Korea). Dulbecco’s phosphate-buffered saline (DPBS), Dulbecco’s modified Eagle medium (DMEM), fetal bovine serum (FBS), penicillin, and streptomycin were purchased from Thermo Fisher Scientific, Inc. (Waltham, MA, USA). A cell proliferation kit (3-(4,5-dimethylthiazol-2-yl)-2,5-diphenyltetrazolium bromide, MTT) was obtained from Roche Diagnostics (Mannheim, Germany). A 2′,7′-dichlorofluorescein diacetate (DCFDA) assay kit for the detection of cellular ROS was obtained from Abcam, Inc. (Cambridge, MA, USA).

### 2.2. Synthesis of MS-Coated rGO

MS-rGO was synthesized according to a previously reported method with minor modifications [44]. After ultrasonicating a GO solution (50 mL) for 30 min, CTAB (0.5 g) and NaOH (20 mg) were sequentially added and vigorously stirred at 40 °C for 1 h. To form a mesoporous structure on a GO surface, 250 μL of TEOS was added dropwise and subsequently reacted 40 °C for 8 h. The mesoporous silica-coated GO (MS-GO) was washed by centrifugation (14,000 rpm, 10 min) and subsequently washed several times with ethanol (EtOH) and deionized water (DW). The remaining CTAB in the nanocomposites was completely removed by using a 10 wt% NaCl solution (50 mL) and washed three times with methanol (MeOH). The reduction process of MS-GO (100 mg) was carried out by treating with hydrazine monohydrate solution (50 mL, 0.16% *v*/*v*), followed by heating to 95 °C for 12 h. The MS-rGO nanocomposite was dialyzed by a molecular weight cut off (MWCO: 3.5 KDa) dialysis membrane in DW for 2 days, and the final product was subsequently lyophilized for 48 h. To introduce amino groups in MS-rGO, MS-rGO (50 mg) dissolved in toluene was sonicated for 10 min. Afterward, APTES (0.5 mL) was slowly added to the MS-rGO solution and stirred at 110 °C for 12 h. Aminated MS-rGO was centrifuged and then rinsed three times with EtOH to remove unreacted APTES. The MS-rGO-NH_2_ was placed into a drying oven at 80 °C for 24 h.

### 2.3. Preparation of MS-rGO-FA and ICG-Encapsulated MS-rGO-FA Nanocomposites

To enhance the colloidal stability and tumor-targeting ability of MS-rGO-NH_2_, FA-PEG-COOH was covalently conjugated to the amino group using EDC/NHS chemistry, as previously described [45]. FA-PEG-COOH (10 mg) dissolved in DMSO was ultrasonicated for 30 min. EDC (50 mg) and NHS (50 mg) were added to the FA-PEG-COOH solution. After 1 h, MS-rGO-NH_2_ (20 mg) was added and stirred for 18 h. To remove unreacted FA-PEG-COOH, MS-rGO-FA was purified using a dialysis membrane (MWCO: 6–8 kDa) and freeze-dried for 48 h. As a control, to compare the tumor targeting ability of nanocomposite, mPEG-COOH (without FA) was anchored on MS-rGO-NH_2_ (MS-rGO-mPEG) in the same manner. To provide NIR-mediated anticancer effects, ICG photosensitizers were encapsulated into MS-rGO-mPEG and MS-rGO-FA nanocomposites, respectively. The MS-rGO-FA, MS-rGO-mPEG (20 mg), and ICG (10 mg) were clearly dispersed in DW and vigorously stirred for 24 h. The unencapsulated ICG was removed by centrifugation (14,000 rpm, 10 min) and washed with DW. ICG-encapsulated MS-rGO-FA (ICG@MS-rGO-FA) and MS-rGO-mPEG (ICG@MS-rGO-mPEG) nanocomposites were obtained by freeze-drying for 48 h.

### 2.4. Characterization of ICG@MS-rGO-FA Nanocomposites

The size and morphology of ICG@MS-rGO-FA nanocomposites were examined using transmission electron microscopy (TEM, JEOL-2100F, JEOL, Tokyo, Japan). The elemental analysis of nanocomposites was confirmed by energy dispersive X-ray spectroscopy (EDX). The surface modification and chemical properties of nanocomposites were confirmed by Fourier transform infrared spectroscopy (FT-IR). FT-IR spectra were recorded by using a Nicolet iS50 instrument (Thermo Fisher Scientific Inc., Waltham, MA, USA), and the surface charge of MS-rGO-FA nanocomposites was measured using a Zetasizer Nano Z (Malvern Instruments, Malvern, UK). To determine the pore size and surface area of nanocomposites, Brunauer-Emmett-Teller (BET) and Barrett-Joyner-Halenda (BJH) analyses were performed by nitrogen gas adsorption-desorption isotherms using an automatic adsorption apparatus, ASAP 2020 analyzer (Micromeritics, Inc., Narcross, GA, USA). The encapsulated ICG into nanocomposites was confirmed by UV-vis spectroscopy (UV 1800, Shimazu, Kyoto, Japan).

### 2.5. Photothermal Performance of ICG@MS-rGO-FA Nanocomposites

The photothermal properties of ICG@MS-rGO-FA nanocomposites were investigated by an 808 nm NIR laser irradiation (MDL-N-808, CNI Optoelectronics Tech. Co., Ltd., Changchun, China). MS-rGO-FA and ICG@MS-rGO-FA nanocomposites dissolved in DW with various concentrations (0.25, 0.5, and 1 mg/mL) were prepared and subsequently irradiated by an 808 nm laser for 10 min with a power density of 1 W/cm^2^. Additionally, the temperature changes via NIR laser irradiation were visualized for predetermined times (10 min) by an infrared thermal imaging system (E60, FLIR Systems, Inc., Wilsonville, OR, USA). To investigate the photothermal stability, ICG@MS-rGO-FA solution was exposed for 10 min, and the following natural cooling process was performed three times. During NIR laser irradiation, the temperature of each solution was measured by thermocouple linked to a digital thermometer (DTM-318, TECPEL Co., Ltd., Taiwan) every 60 sec for 10 min.

### 2.6. Detection of ROS from ICG@MS-rGO-FA Nanocomposites

The generation of ROS from ICG@MS-rGO-FA nanocomposites was confirmed by DPBF probe [46]. Briefly, 10 μL DPBF solution in DMSO (1 mg/mL) was prepared and mixed with 200 μL of ICG@MS-rGO-FA solution (0.1 mg/mL). The mixture solution was irradiated with an 808 nm NIR laser (1 W/cm^2^) for different time periods (0, 2, 4, 6, 8, and 10 min). All processes were carried out in a dark environment. As a control, a MS-rGO-FA nanocomposite was also subjected to the same protocol. The corresponding absorption curves of DPBF were obtained by UV-vis spectroscopy (410 nm).

### 2.7. Cytotoxicity Analysis of ICG@MS-rGO-FA Nanocomposites

The cytotoxicity of ICG@MS-rGO-FA nanocomposites was evaluated by an MTT assay. NIH-3T3 and CT-26 cells were seeded in a 96-well plate at a density of 1 × 10^4^ cells per well and incubated for 24 h at 37 °C. Then, 100 μL of ICG@MS-rGO-FA nanocomposites at various concentrations (10–100 μg/mL) were added to each well, and the plate was subsequently incubated for 24 h. The cells were rinsed with DPBS, and the medium was replaced with fresh culture medium containing an MTT agent (0.5 mg/mL). After incubation for 4 h, the medium was carefully removed, and 200 μL of DMSO was added to each well to dissolve the internalized purple formazan crystals. The absorption was measured at 570 nm using an iMark^TM^ microplate reader (Bio-Rad, Hercules, CA, USA).

### 2.8. Cellular Uptake Analysis of ICG@MS-rGO-FA Nanocomposites

In NIH-3T3 fibroblast and CT-26 cells, the cellular uptake and endocytosis pathways of nanocomposites were evaluated by using confocal laser scanning microscopy (CLSM, LSM 880, Carl Zeiss, Jena, Germany). The cells were seeded in 8-well plates (ibidi, Munich, Germany) at a density of 2 × 10^4^ cells/mL. The cells were incubated with ICG@MS-rGO-FA nanocomposites (100 μg) in 1 mL serum-free medium for 4 h. Subsequently, the cells were washed three times with DPBS to remove the remaining nanocomposites and then fixed with 4% paraformaldehyde for 15 min. After treatment with 0.1% Triton X-100 for 10 min at room temperature, the cells were stained with Alexa Fluor 488 phalloidin (1:200, Invitrogen, Waltham, MA, USA) for 1 day at 4 °C and 4′,6-diamidino-2-phenylindole (DAPI, Thermo Fisher Scientific, Waltham, MA, USA) for 5 min. The ICG@MS-rGO-mPEG nanocomposites were used as a control sample, and cellular uptake was also observed with the same protocol.

### 2.9. Detection of Intracellular ROS via NIR Laser Irradiation

The intracellular generation of ROS in CT-26 cells was performed by using a DCFDA assay. CT-26 cells (2 × 10^4^ cells/mL) were seeded and subsequently cultured for 24 h. MS-rGO-FA and ICG@MS-rGO-FA nanocomposites (100 μg/mL) were treated with CT-26 cells for 12 h. Each cell was exposed to an 808 nm NIR laser for 5 min at a power density of 3 W/cm^2^ and then washed several times with DPBS. DCFDA (50 μM) was added to each well, which was then incubated for 40 min. To evaluate the generation of intracellular ROS via nanocomposites, the fluorescent signal at 525 nm was observed by using inverted fluorescence microscopy (Olympus IX73, Tokyo, Japan).

### 2.10. Synergistic In Vitro Anticancer Effect of ICG@MS-rGO-FA Nanocomposites

The improved therapeutic effect of ICG@MS-rGO-FA nanocomposites was demonstrated by an MTT assay. CT-26 cells were seeded in a 96-well plate (1 × 10^4^ cells) and incubated for 24 h. The cells were treated with ICG@MS-rGO-FA nanocomposites at various concentrations (0–140 μg/mL). After incubation for 12 h, CT-26 cells were irradiated with or without an NIR laser (808 nm, 3 W/cm^2^) for 5 min. CT-26 cells were also washed with DPBS and MTT solution was added into each well. After 4 h, DMSO was added and the absorbance was measured by using a microplate reader. Additionally, a live and dead assay was performed to visualize the anticancer effect under an NIR light irradiation. The ICG@MS-rGO-FA nanocomposites treated with CT-26 cells were incubated for 4 h and subsequently irradiated with an NIR laser for 5 min. The CT-26 cells were stained with calcein AM and ethidium homodimer-1. After 30 min, the cells were washed several times with DPBS. Live and dead images were obtained by using inverted fluorescence microscopy.

### 2.11. In Vivo and Ex Vivo Biodistribution Analysis

Female Balb/c mice (6–8 weeks old) were used for animal experiments in this study. To establish the tumor xenograft model, 1 × 10^7^ CT-26 cells in 100 µL of PBS were subcutaneously injected into the right flank region of the mice. When the tumor volume reached approximately 50–80 mm^3^, the following in vivo experiments were performed. Tumor volume was calculated as (tumor length) × (tumor width)^2^/2. For the in vivo biodistribution assay, CT-26 tumor-bearing mice were randomly divided and intravenously injected with 100 µL of ICG@MS-rGO-mPEG and ICG@MS-rGO-FA (2.5 mg/kg, *n* = 4 per group). Mice were imaged in a time-dependent manner with an in vivo imaging system (IVIS) imager (Lumina XRMS, PerkinElmer, Waltham, MA, USA). After the completion of in vivo imaging at 24 h, all mice were sacrificed, and their major organs were excised for ex vivo imaging and imaged with an IVIS imager. All imaging analyses were processed with IVIS imaging software (Living Imaging, PerkinElmer, Waltham, MA, USA). For fluorescence imaging of tissue sections, the dissected tumors were completely frozen in OCT (cryo-embedding media) at −80 °C and cut into 5-µm-thick sections. The tumor sections were stained with DAPI and observed by CLSM.

### 2.12. In Vivo Infrared Thermal Imaging and Anticancer Therapy

CT-26 tumor-bearing mice were divided into four groups (saline, MS-rGO-FA, ICG@MS-rGO-mPEG, and ICG@MS-rGO-FA nanocomposites) and intravenously injected with 100 µL of each sample based on a previously published similar dose range for nanocomposites (2.5 mg/kg, *n* = 6 per group) [47,48]. The mice were repetitively irradiated in the tumor region by an 808 nm laser (2.0 W/cm^2^) for 10 min at 4 h, 24 h, and 48 h after i.v. injection of each sample. Then, the infrared thermal images were recorded by a thermal imaging camera (E60, FLIR Systems Inc., Wilsonville, OR, USA, Sweden). At 10 days after sample injection, all mice were sacrificed to collect major organs and blood. The obtained tissues were fixed in 4% paraformaldehyde, embedded in paraffin, cut into 5-µm-thick sections, and then stained with hematoxylin and eosin (H&E). For biochemical analysis, the collected blood samples were centrifuged at 3000 rpm at 4 °C for 15 min to obtain serum. The serum levels of alanine aminotransferase (ALT), aspartate aminotransferase (AST), creatinine, and uric acid were measured to evaluate renal and liver function.

## 3. Results and Discussion

### 3.1. Synthesis and Characterization of ICG@MS-rGO-FA Nanocomposites

**Scheme 1:**
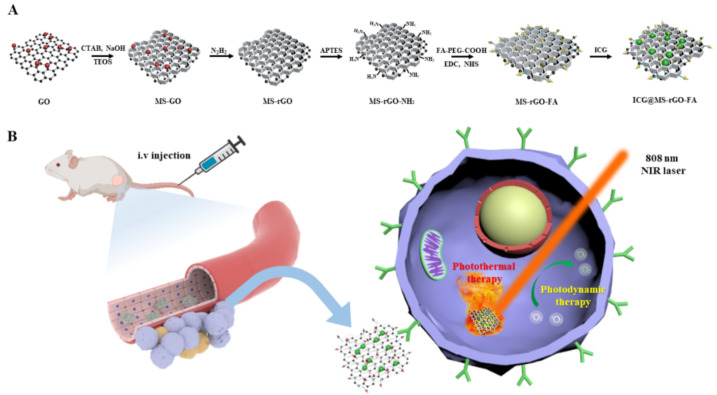
(**A**) Synthetic procedure of tumor targeted ICG-encapsulated MS-rGO-FA nanocomposites and (**B**) application for enhanced PTT and PDT dual therapy in colorectal cancer.

To obtain the MS-coated rGO nanocomposites (MS-rGO), GO nanosheets were coated with MS as a single layer via sol-gel condensation with silica precursor (TEOS) and cationic surfactant (CTAB) and then reduced by hydrazine monohydrate, as previously described [49]. MS-rGO-FA nanocomposites were subsequently fabricated with PEG-FA (5 kDa) by EDC/NHS chemical reaction, which led to colloidal stability of nanocomposites and their selective targeting of folate receptor-positive cancer cells. Finally, ICG molecules were encapsulated on MS-rGO-FA nanocomposites by using hydrophobic and π-π stacking interactions (Scheme 1).

**Figure 1:**
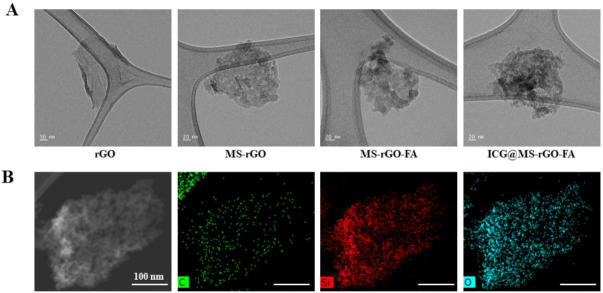
(**A**) TEM images of rGO, MS-rGO, MS-rGO-FA, and ICG@MS-rGO-FA nanocomposites. (**B**) EDX mapping of MS-rGO nanocomposites.

The size, morphology, and elemental analysis of nanocomposites were evaluated by TEM. As shown in Figure 1A, MS-rGO, MS-rGO-FA, and ICG@MS-rGO-FA nanocomposites formed a uniform sphere shape, whereas rGO nanosheet showed a transparent single or double nanosheet structure due to the spontaneous aggregation of rGO [44]. The average diameters of the MS-rGO, MS-rGO-FA, and ICG@MS-rGO-FA nanocomposites were approximately 165.7 ± 7.2, 175.5 ± 8.5, and 191.7 ± 9.2 nm, respectively. According to the elemental mapping results in Figure 1B, we confirmed that the main elements of the MS-rGO nanocomposites were Si and O, indicating successful fabrication of MS on the rGO nanosheet. Figure S1 represented the diameter of nanocomposites in an aqueous solution, showing that the size of the ICG@MS-rGO-FA nanocomposites was larger than that of the MS-rGO and MS-rGO-FA nanocomposites. These results revealed the successful synthesis of ICG-encapsulated MS-rGO-FA nanocomposites.

**Figure 2:**
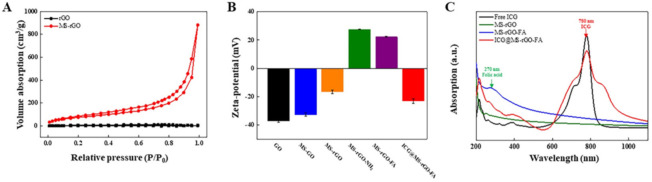
The physicochemical properties of ICG@MS-rGO-FA nanocomposites. (**A**) N_2_ adsorption-desorption isotherm of rGO and MS-rGO nanocomposites. (**B**) Zeta-potential of ICG@MS-rGO-FA nanocomposites. (**C**) UV-vis spectra of free ICG, MS-rGO-mPEG, MS-rGO-FA, and ICG@MS-rGO-FA nanocomposites.

The surface area, pore volume, and pore size of MS-rGO nanocomposites were characterized by BET-BJH analysis (Figure 2A). The surface area and pore volume of the pure rGO nanosheet could not be calculated because they did not show N_2_ adsorption-desorption. On the other hand, the MS-rGO exhibited a large surface area (283.05 m^2^/g), pore volume (1.33 cm^3^/g), and pore size (3.8 nm). The N_2_ adsorption-desorption isotherm curve indicated that the MS-rGO nanocomposites were composed of well-defined mesoporous structures and small pore size distributions [44]. The surface charge and chemical construction of ICG@MS-rGO-FA nanocomposites were confirmed by zeta potential, FT-IR, and UV-vis spectroscopy. In Figure 2B, the zeta potentials of the MS-GO, MS-rGO, MS-rGO-NH_2_, MS-rGO-FA, and ICG@MS-rGO-FA nanocomposites were −32.6 ± 1.3, −16.5 ± 1.3, +27.5 ± 0.2 mV, +22.2 ± 0.5, and −23 ± 1.6 mV, respectively. These changes in the zeta potential implied the successful reduction, introduction of amine groups, conjugation of PEG-FA, and encapsulation of ICG molecules [45,50]. Figure S2 shows the FT-IR spectra of the MS-rGO and MS-rGO-FA nanocomposites. The strong peaks at 962 and 1074 cm^−1^ corresponding to the stretching vibration of Si-O-Si were attributed to the MS monolayer. The chemical conjugation of PEG-FA on MS-rGO nanocomposites was confirmed by the new characteristic peaks. The peaks of PEG were observed at 1350 and 1450 cm^−1^ due to the -CH_2_ and CH_3_ framework stretching in PEG. It also showed -C-O-C- asymmetrical and symmetrical stretching at 955 and 1100 cm^−1^ [45,50]. However, FA, the tumor-targeting ligand, was not detected by FT-IR analysis because its structure was similar to that of the PEG molecule [45]. Therefore, the presence of FA and encapsulated ICG was demonstrated by UV-vis spectroscopy measurement, showing that the spectra of MS-rGO nanocomposites did not show any absorption peak at 280 nm (Figure 2C). In contrast, MS-rGO-FA and ICG@MS-rGO-FA nanocomposites exhibited additional peaks in the same area. In the spectra of ICG@MS-rGO-FA nanocomposite, two strong peaks were observed at 710 and 780 nm. The new peaks at 280 nm and 700–800 nm indicated the successful conjugation of FA and encapsulation of ICG in the nanocomposites [51,52,53]. Additionally, we calculated the loading efficiency of ICG in the rGO nanocomposites with or without MS based on the UV-vis absorption spectra (at 780 nm). We observed that the loading efficiency of ICG in rGO-FA nanocomposites was only 6% in the absence of MS. In contrast, MS-rGO-FA nanocomposites showed approximately 22% loading efficiency, suggesting that the mesoporous structure of rGO nanosheet significantly improved the loading capacity of ICG molecules [54].

### 3.2. Enhanced Photothermal Performance of ICG@MS-rGO-FA Nanocomposites

**Figure 3:**
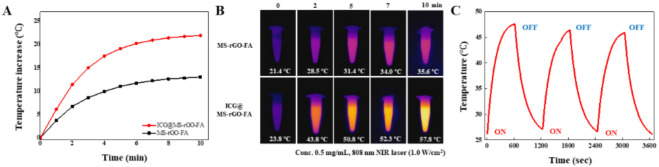
Photothermal performance of nanocomposites. (**A**) Temperature curve and (**B**) IR thermal images of MS-rGO-FA and ICG@MS-rGO-FA (0.5 mg/mL) nanocomposites exposed to an 808 nm NIR laser (1.0 W/cm^2^). (**C**) Photostability of ICG@MS-rGO-FA nanocomposites after three ON/OFF cycles of an 808 nm NIR laser irradiation (1.0 W/cm^2^, 10 min).

The PTT performance of ICG@MS-rGO nanocomposites was verified by temperature change by an 808 nm NIR laser irradiation (Figure 3). We evaluated the PTT properties of MS-rGO-FA and ICG@MS-rGO-FA nanocomposites (Figure 3A). The temperature of MS-rGO-FA increased only to 12.9 °C, while ICG@MS-rGO-FA nanocomposites elevated up to 21.9 °C under the same experimental condition (200 μL, 0.5 mg/mL in DW, and 1.0 W/cm^2^). Furthermore, the IR thermal images were obtained for visualization of temperature changes (Figure 3B). After irradiation for 10 min, the color in the thermal image of MS-rGO-FA nanocomposites was changed from violet to purple. In the case of ICG@MS-rGO-FA nanocomposites, the color was shifted to yellow. Through IR thermal images, it was confirmed that the temperature of ICG@MS-rGO-FA was 1.7-fold increased as compared to that of MS-rGO-FA nanocomposites, corresponding to the PTT effect in Figure 3A. These results demonstrated that the enhanced PTT performance was derived via combination of encapsulated ICG in MS-rGO-FA and inherent PTT effect of rGO nanosheet. Additionally, we investigated ICG@MS-rGO-FA nanocomposites at different concentrations (0.25–1.0 mg/mL) with an NIR laser (Figure S3). As expected, the nanocomposites exhibited a temperature rise by 14.9, 21.9, and 26 °C, respectively, depending on the concentrations of nanocomposites. We subsequently observed the photostability of ICG@MS-rGO-FA nanocomposites in Figure 3C. Although NIR laser irradiation was repetitively performed at least 3 times, the temperature was steadily increased to 46 °C without any thermal loss, showing that the ICG-encapsulated MS-rGO-FA nanocomposite could be a suitable PTT candidate for cancer treatment.

### 3.3. Photodynamic Properties of ICG@MS-rGO-FA Nanocomposites

To investigate the photodynamic therapy of ICG@MS-rGO-FA nanocomposites via NIR laser irradiation, DPBF probes were applied for the detection of ROS from ICG-encapsulated nanocomposites (Figure S4). The ICG@MS-rGO-FA nanocomposites and DPBF probes were mixed and subsequently irradiated with an NIR laser every 2 min. In parallel, the control groups (only DPBF probe and ICG-free MS-rGO-FA nanocomposites) were treated in the same manner. In Figure S4A, the absorption intensity of ICG-encapsulated nanocomposites at 417 nm was inversely proportional to the exposure time of NIR light because DPBF probes were reacted with generated ROS from ICG@MS-rGO-FA nanocomposites [55]. In contrast, the control groups showed constant intensity regardless of NIR laser irradiation, suggesting that the generated ROS was insufficient (Figure S4B). Therefore, the excellent ROS generation capacity and enhanced photothermal property of ICG@MS-rGO-FA nanocomposites have great potential for NIR-mediated phototherapy applications.

### 3.4. In Vitro Cytotoxicity and Cellular Uptake Analysis of ICG@MS-rGO-FA Nanocomposites

**Figure 4:**
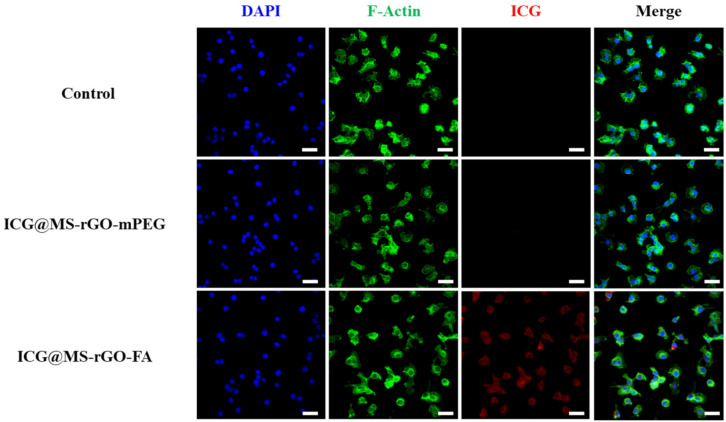
Cellular uptake of ICG@MS-rGO-mPEG and ICG@MS-rGO-FA nanocomposites to CT-26 cancer cells. Scale bars are 20 μm. DAPI: blue, ICG: red color.

The biocompatibility and low toxicity of GO-based biomaterials are of great importance to biological applications. Therefore, the cytotoxicity of the ICG@MS-rGO-FA nanocomposites should be assessed prior to performing in vivo experiments. The potential cytotoxicity of ICG@MS-rGO-FA nanocomposites was evaluated by using NIH-3T3 fibroblast cells and CT-26 CRC cells for the correlative experimentation. Figure S5 showed the cell viability of NIH-3T3 fibroblast cells and CT-26 cells cultured with the ICG-encapsulated MS-rGO-FA nanocomposites at various concentrations for 24 h by MTT assay. After 24 h incubation, the proportion of viable cells treated with ICG@MS-rGO-FA nanocomposites did not decrease to below 85%, even at a high concentration of nanocomposites (100 μg/mL). In addition, as a control, MS-rGO-FA nanocomposites were treated with NIH-3T3 fibroblast cells and CT-26 cells. The cell viability was observed in the same procedure. Similar to the previous results, NIH-3T3 fibroblast cells and CT-26 cells exhibited high viabilities at various concentrations of nanocomposites (>85%, 0–100 μg/mL). These results revealed that ICG-encapsulated MS-rGO-FA and ICG-free MS-rGO-FA nanocomposites not only exhibited excellent biocompatibility but might also be suitable for clinical applications [56]. We further investigated the cellular uptake of ICG@MS-rGO-FA nanocomposites by confocal laser scanning microscopy, showing that intracellular nanocomposites were observed in folate receptor-negative NIH-3T3 fibroblast cells and positive CT-26 cancer cells (Figure 4 and Figure S6). After 4 h of incubation with ICG@MS-rGO-FA nanocomposites, CT-26 cells presented a red fluorescent signal in the cytoplasm. In contrast, CT-26 cells treated with ICG@MS-rGO-mPEG nanocomposites and NIH-3T3 fibroblast cells treated with ICG@MS-rGO-FA nanocomposites showed no fluorescence intensity. Therefore, these results indicated that the ICG@MS-rGO-FA nanocomposites were internalized in CT-26 cells via the FR-mediated endocytosis pathway, as previously described [57].

### 3.5. Intracellular ROS Detection via NIR Laser Irradiation

**Figure 5:**
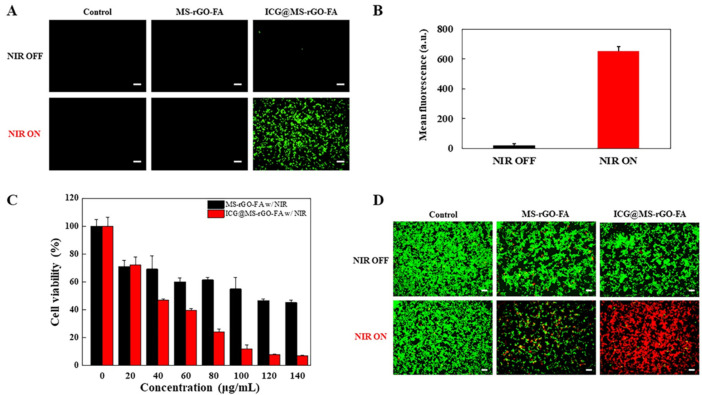
(**A**) Detection of intracellular ROS in CT-26 cancer cells treated with MS-rGO-FA and ICG@MS-rGO-FA nanocomposites, with or without NIR laser irradiation. Scale bars are 50 μm. (**B**) Mean fluorescence intensity of generated ROS in CT-26 cells with ICG@MS-rGO-FA nanocomposites, with or without NIR laser irradiation. (**C**) Quantitative analysis of the viability of CT-26 cells treated with MS-rGO-FA and ICG@MS-rGO-FA nanocomposites, with 808 nm NIR laser irradiation. (**D**) Fluorescence images of live/dead assay in CT-26 cells after treatment of MS-rGO-FA and ICG@MS-rGO-FA nanocomposites (100 μg/mL), with or without NIR laser irradiation. The live and dead cells were stained with calcein AM (green) and ethidium homodimer-1 (red). Scale bars are 50 μm.

To confirm the generation of ROS in CT-26 cells with or without NIR laser irradiation, the mixture of nanocomposites and DCFDA probe was treated with CT-26 cells [46]. As shown in Figure 5A, when CT-26 cells treated with MS-rGO-FA and ICG@MS-rGO-FA nanocomposites were not exposed to NIR laser irradiation, ROS were not detected in CT-26 cells. However, after incubation with ICG@MS-rGO-FA nanocomposites and treatment with NIR laser irradiation, the green fluorescent signal largely spread throughout the CT-26 cells, indicating the successful generation of intracellular ROS via nanocomposites. Furthermore, we analyzed the amount of the generated ROS in CT-26 cells. Figure 5B showed the relative fluorescence intensity of ROS generated from CT-26 cells treated with ICG@MS-rGO-FA nanocomposites, with or without NIR laser irradiation. The mean intensity under NIR laser irradiation was increased 30-fold, suggesting that ICG@MS-rGO-FA nanocomposites could be used for tumor-targeted PDT applications.

### 3.6. Synergistic Therapeutic Effects with Enhanced PTT and PDT Treatment

To demonstrate NIR-mediated therapeutic effects via enhanced PTT and PDT treatment, CT-26 cancer cells were cultured with ICG@MS-rGO-FA nanocomposites for 24 h, and in vitro cytotoxicity was evaluated by an MTT assay (Figure 5C). Upon NIR laser irradiation, the cell viability treated with MS-rGO-FA nanocomposites decreased up to 45% as the concentration was increased. This reduced value was derived from the rGO nanosheet-mediated PTT effect [45]. Compared to PTT alone, the enhanced PTT and PDT based on ICG-encapsulated nanocomposites resulted in excellent NIR laser-mediated therapeutic efficacy. When ICG@MS-rGO-FA nanocomposites (100 μg/mL) were treated with CT-26 cells under NIR laser irradiation, the cell viability was dramatically decreased, and the cells were only 10% viable. To prove the synergistic therapeutic efficacy of enhanced PTT and PDT in CT-26 cells, a live and dead assay was performed (Figure 5D). In the control groups, most CT-26 cells exhibited strong green fluorescence, regardless of NIR laser irradiation, indicating that NIR laser irradiation was not harmful to the tumor cells [58]. In CT-26 cells treated with MS-rGO-FA nanocomposites and exposed to NIR laser irradiation, a few sporadic red fluorescence regions were observed because rGO nanosheets showed the PTT effect on cancer cells under NIR laser irradiation. In CT-26 cells containing ICG@MS-rGO-FA nanocomposites, most CT-26 cells were dead, confirming that our NIR-mediated nanocomposites could effectively eradicate CT-26 CRC cells.

### 3.7. In Vivo Biodistribution of ICG@MS-rGO-FA Nanocomposites

**Figure 6:**
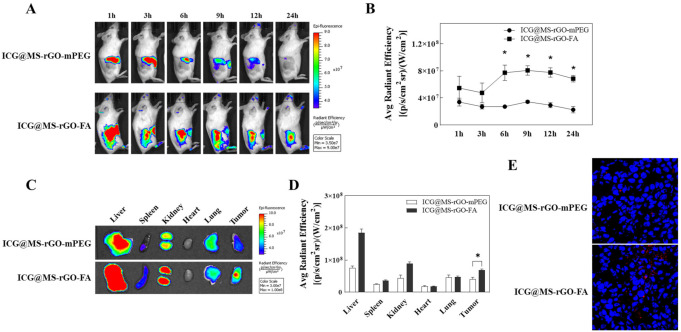
In vivo biodistribution imaging of ICG@MS-rGO-mPEG and ICG@MS-rGO-FA nanocomposites in CT-26 tumor-bearing mice. (**A**) Representative in vivo fluorescence imaging after i.v. injection at different times (1 h, 3 h, 6 h, 9 h, 12 h, and 24 h). (**B**) Fluorescence intensity of CT-26 tumor tissues (*n* = 4, * *p* < 0.05). (**C**) Representative ex vivo fluorescence imaging of major organs at 24 h post-injection. (**D**) Fluorescence intensity of major organs (*n* = 4, * *p* < 0.05). (**E**) Fluorescence microscopy imaging of tumor tissues.

To investigate in vivo biodistribution and tumor targeting capability, CT-26 tumor-bearing mice were intravenously treated with ICG@MS-rGO-mPEG and ICG@MS-rGO-FA nanocomposites. The in vivo NIR fluorescence imaging was performed to monitor the time-dependent changes in the ICG fluorescence signal. As shown in Figure 6A,B, ICG@MS-rGO-FA nanocomposites resulted in high tumor accumulation and were retained in the tumors until 24 h post-injection. In contrast, we observed the low fluorescence signals of ICG@MS-rGO, indicating that ICG@MS-rGO-mPEG nanocomposites without a targeting moiety were limited to targeting specific tumors. After 24 h, ex vivo fluorescence imaging exhibited significantly higher tumor accumulation in ICG@MS-rGO-FA nanocomposites-treated mice than in ICG@MS-rGO-mPEG nanocomposites-treated mice, further indicating the enhanced tumor targeting capability of ICG@MS-rGO-FA nanocomposites (Figure 6C,D). In addition, the fluorescence microscopy imaging of tumor tissues revealed a substantial increase in the cellular distribution in the tumors treated with ICG@MS-rGO-FA nanocomposites (Figure 6E), which were highly consistent with in vitro cellular uptake and in vivo biodistribution (Figure 4 and Figure 6A,C). These results suggested that ICG@MS-rGO-FA nanocomposites could enhance in vivo tumor targeting and uptake associated with folate receptor-mediated endocytosis.

### 3.8. In Vivo Synergistic Anticancer Effects of PTT and PDT

**Figure 7:**
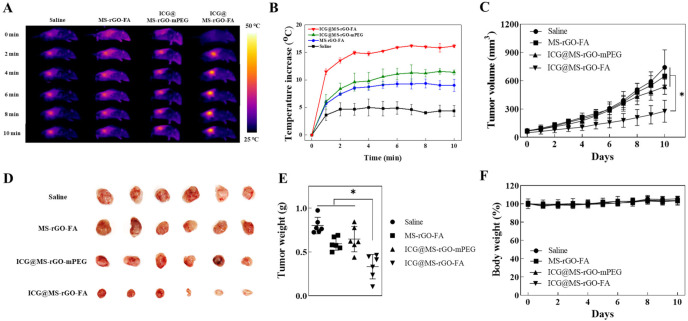
In vivo anticancer therapy. (**A**) IR thermal images of CT-26 tumor-bearing mice that received different treatments. (**B**) Temperature curves of tumors in mice of different. (**C**) Tumor growth curve with different treatments. (**D**) Photograph showing the tumor from mice with various treatments. (**E**) Tumor weight from each group of the mice. (**F**) Body weight of mice in the various administrations. * *p* < 0.05.

To evaluate the combined therapeutic effects via enhanced PTT and PDT in vivo, the temperature changes in the tumor region were monitored using an infrared thermal imaging system. CT-26 tumor-bearing mice were treated with saline, MS-rGO-FA, ICG@MS-rGO-mPEG, and ICG@MS-rGO-FA nanocomposites, followed by NIR laser irradiation for 10 min. During NIR laser irradiation, ICG@MS-rGO-FA nanocomposites led to significantly higher temperature increases in the tumors than those of MS-rGO-FA and ICG@MS-rGO-mPEG nanocomposites (Figure 7A,B), which evidenced the enhanced effect of ICG@MS-rGO-FA nanocomposites via PTT and PDT in the targeted tumor region. In addition, the in vivo anticancer efficacy of ICG@MS-rGO-FA nanocomposite was further evaluated by repeated NIR laser irradiation. CT-26 tumors treated with saline, MS-rGO-FA, and ICG@MS-rGO-mPEG nanocomposites showed progressive growth, whereas tumors treated with ICG@MS-rGO-FA nanocomposites exhibited significantly delayed growth (Figure 7C–E). These results could be attributed to improved targeting of folate receptor-positive CT-26 CRC cells and their synergistic effects of enhanced PTT and PDT under NIR laser irradiation. To investigate the biocompatibility and potential toxicity of ICG@MS-rGO-FA nanocomposites, the changes in body weight, biochemical markers, and histopathology of the major organs were evaluated (Figure 7F). As expected, there was no significant loss of body weight, as well as no apparent differences in biochemical assessment of renal and liver function, including alanine aminotransferase (ALT), aspartate aminotransferase (AST), creatinine, and uric acid levels (Figure S7). In addition, the histopathological H&E staining of the major organs revealed no abnormal morphologies, indicating that it caused no obvious systematic toxicity in all treatment groups (Figure S8). Taken together, these data indicated that the enhanced tumor targeting of ICG@MS-rGO-FA nanocomposites could effectively suppress the tumor growth by synergistic PTT and PDT effects with excellent biocompatibility.

## 4. Conclusions

We synthesized NIR laser-mediated ICG@MS-rGO-FA nanocomposites for enhanced PTT and PDT cancer therapy. The ICG@MS-rGO-FA nanocomposites provided various advantages, such as improved loading capacity of ICG, excellent targeting ability to FR-positive cancer cells, enhanced photothermal effect, and ROS generation capability underNIR laser irradiation. In vitro studies demonstrated that ICG@MS-rGO-FA nanocomposites showed excellent biocompatibility and improved cellular uptake performance in folate receptor-overexpressing CT-26 cancer cells. The enhanced PTT and PDT therapeutic effects of ICG@MS-rGO-FA nanocomposites were clearly observed by viability of CT-26 cells in vitro. Furthermore, in vivo biodistribution and anticancer studies in CT-26 tumor-bearing mice exhibited that ICG@MS-rGO-FA nanocomposites led to enhanced tumor targeting, with less toxicity to normal tissues and excellent therapeutic efficacy, by synergistic enhanced PTT and PDT effects. Therefore, this ICG@MS-rGO-FA nanocomposite could be a potentially powerful phototherapeutic nanocarrier.

## Data Availability

The data presented in the study are available in this manuscript.

## Supplementary Materials

The following are available online at https://www.mdpi.com/article/10.3390/antiox11010174/s1, Figure S1: Size distribution of MS-rGO, MS-rGO-FA, and ICG@MS-rGO-FA nanocomposites, Figure S2: FT-IR spectra of MS-GO, MS-rGO, and MS-rGO-FA nanocomposites, Figure S3: Temperature analysis of ICG@MS-rGO-FA nanocomposites with various concentrations under NIR laser irradiation (0.25–1.0 mg/mL, 1.0 W/cm^2^), Figure S4: (**A**) Absorption spectra of DPBF solution, including ICG@MS-rGO-FA nanocomposites after different times with 808 nm laser irradiation (1.0 W/cm^2^). (**B**) The ROS generation curves of DPBF probe, MS-rGO-FA, and ICG@MS-rGO-FA nanocomposites, respectively, Figure S5: Cytotoxicity analysis of (**A**) NIH-3T3 fibroblast cells and (**B**) CT-26 cells treated with MS-rGO-FA and ICG@MS-rGO-FA nanocomposites, Figure S6: Confocal fluorescence images of NIH-3T3 fibroblast cells treated with ICG@MS-rGO-FA nanocomposites. Scale bars are 20 μm, Figure S7: Serum biochemical parameters assay (ALT, AST, creatinine, and uric acid), Figure S8: Histological images (H&E staining) obtained in major organs (e.g., liver, lung spleen, kidney, and heart) of the mice after treatment under various conditions.
