# Supplementary material for: Near-Infrared Light-Triggered Generation of Reactive Oxygen Species and Induction of Local Hyperthermia from Indocyanine Green Encapsulated Mesoporous Silica-Coated Graphene Oxide for Colorectal Cancer Therapy
Source: Antioxidants (Basel). 2022 Jan 17;11(1):174. doi: 10.3390/antiox11010174 (PMC8772730; doi:10.3390/antiox11010174)
Supplement: Supplementary file 1 [file antioxidants-11-00174-s001.zip › antioxidants-1541959-supplementary.pdf]

SUPPLEMENTARY MATERIALS

Near-infrared Light Triggered Generation of Reactive Oxygen Species from Indocyanine Green Encapsulated Mesoporous Silica-coated Graphene Oxide for Colorectal Therapy

Hyung Woo Choi[1,†], Jae Hyun Lim[2,†], Chan Woo Kim[3,4,†], Eunmi Lee[3,4], Jin-Moo Kim[3,4], Kiyuk Chang[3,4,*], Bong Geun Chung[1,*]

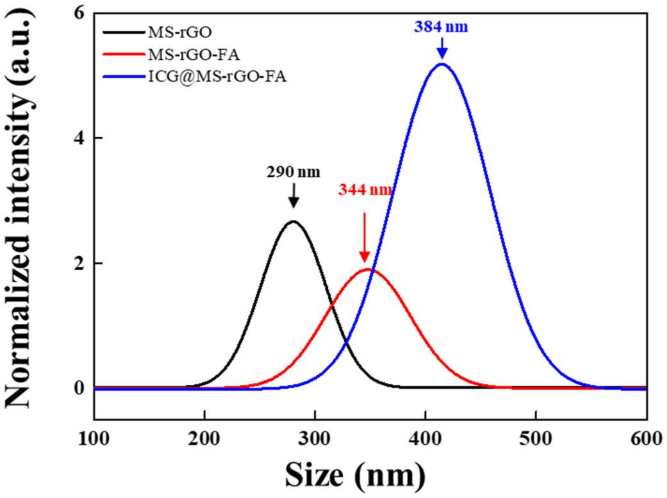

Figure S1. Size distribution of MS-rGO, MS-rGO-FA, and ICG@MS-rGO-FA nanocomposites.

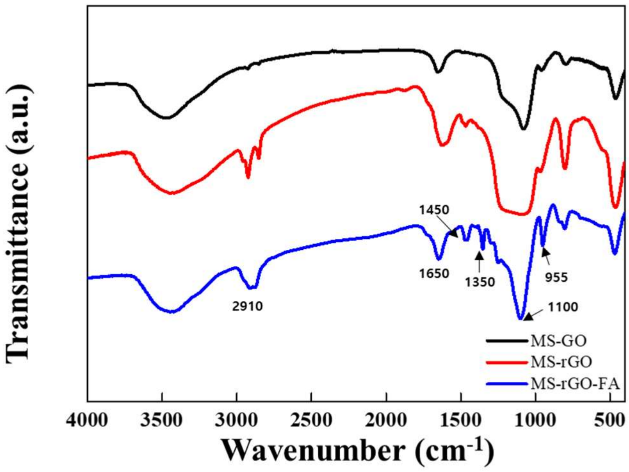

Figure S2. FT-IR spectra of MS-GO, MS-rGO, and MS-rGO-FA nanocomposites.

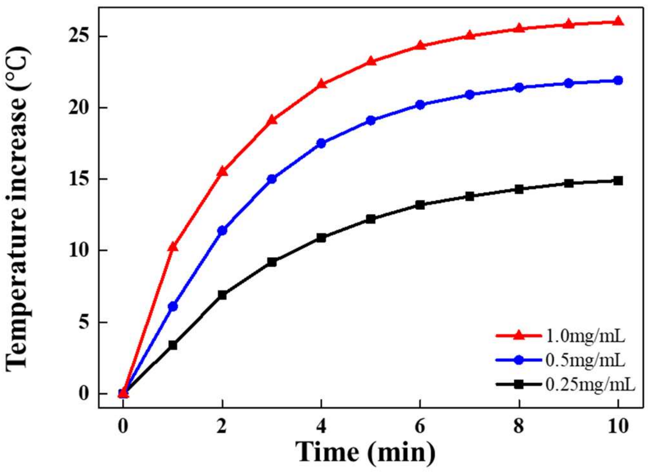

Figure S3. Temperature analysis of ICG@MS-rGO-FA nanocomposites with various concentrations under NIR laser irradiation (0.25-1.0 mg/mL, 1.0 W/cm^2).

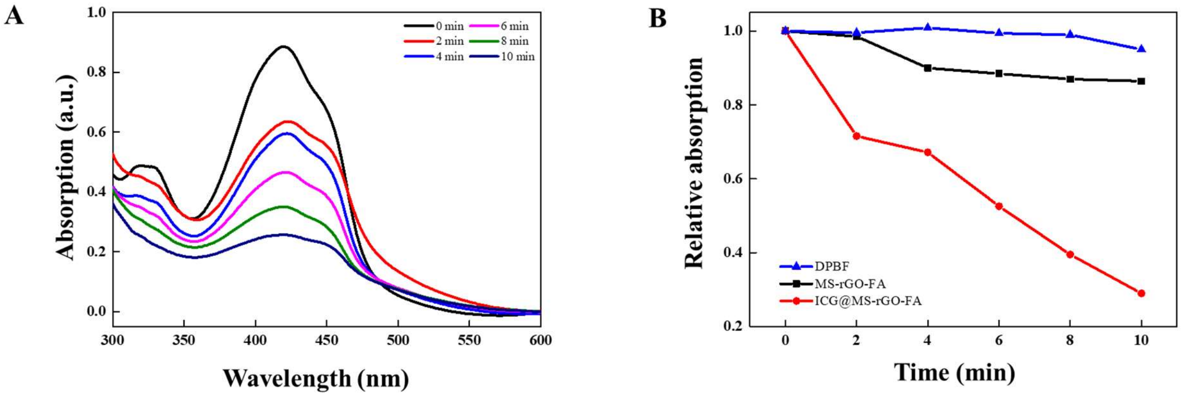

Figure S4. (A) Absorption spectra of DPBF solution including ICG@MS-rGO-FA nanocomposites after different times with 808 nm laser irradiation (1.0 W/cm^2). (B) The ROS generation curves of DPBF probe, MS-rGO-FA, and ICG@MS-rGO-FA nanocomposites, respectively.

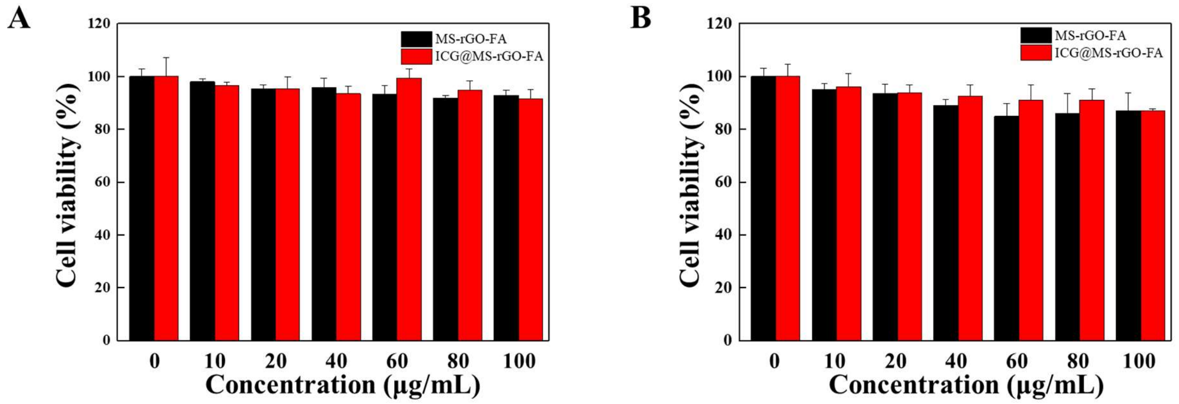

Figure S5. Cytotoxicity analysis of (A) NIH-3T3 fibroblst cells and (B) CT-26 cells treated with MS-rGO-FA and ICG@MS-rGO-FA nanocomposites.

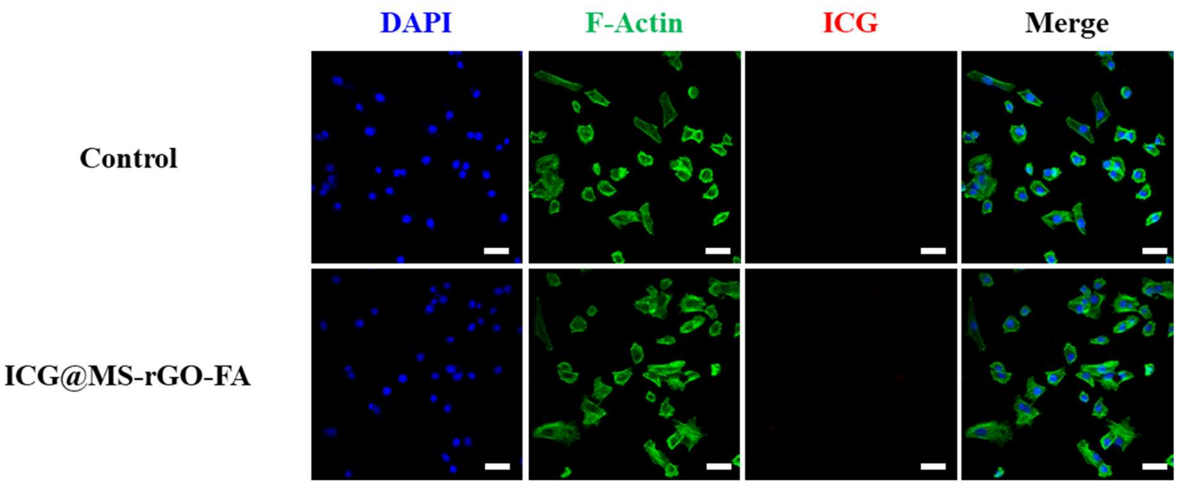

Figure S6. Confocal fluorescence images of NIH-3T3 fibroblast cells treated with ICG@MS-rGO-FA nanocomposites. Scale bars are 20 μm.

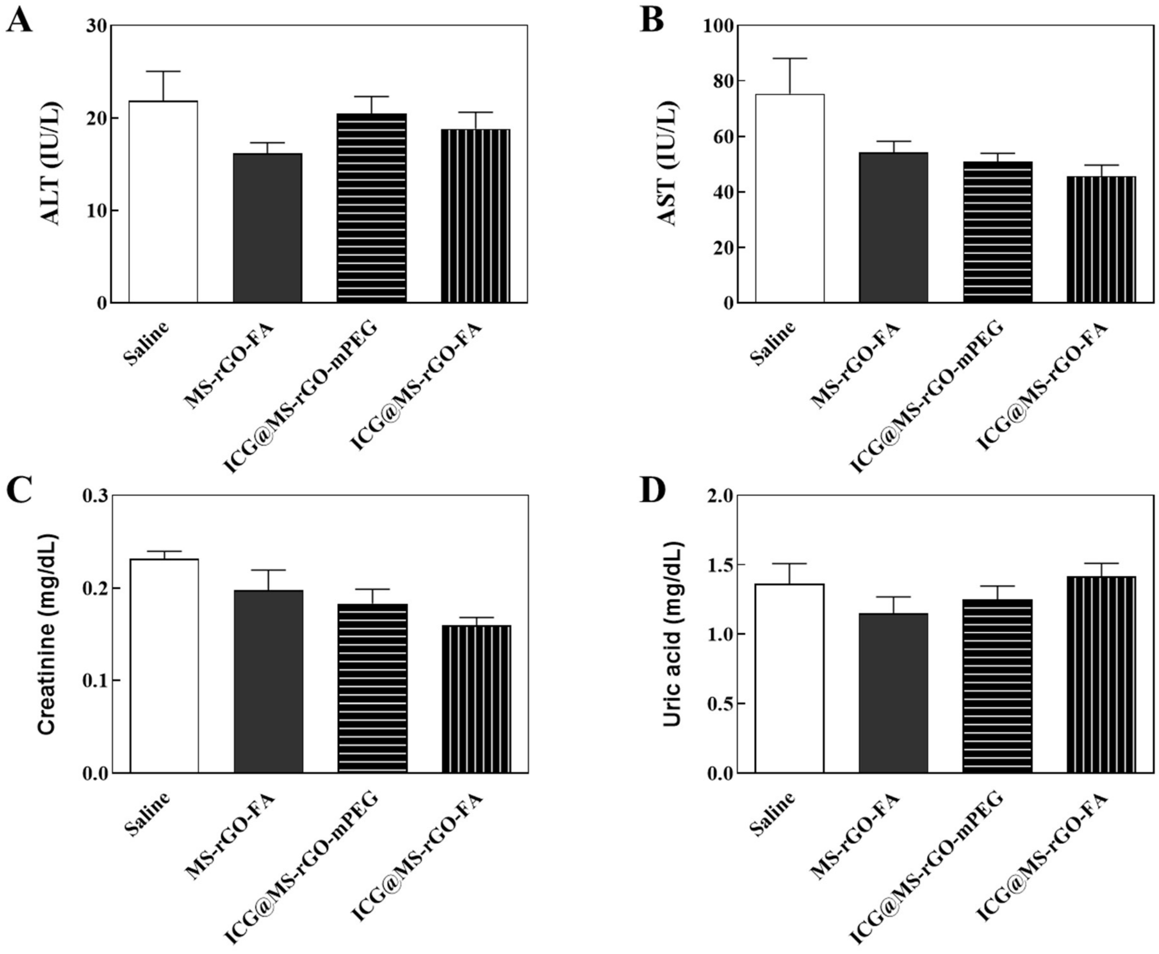

Figure S7. Serum biochemical parameters assay (ALT, AST, creatinine, and uric acid).

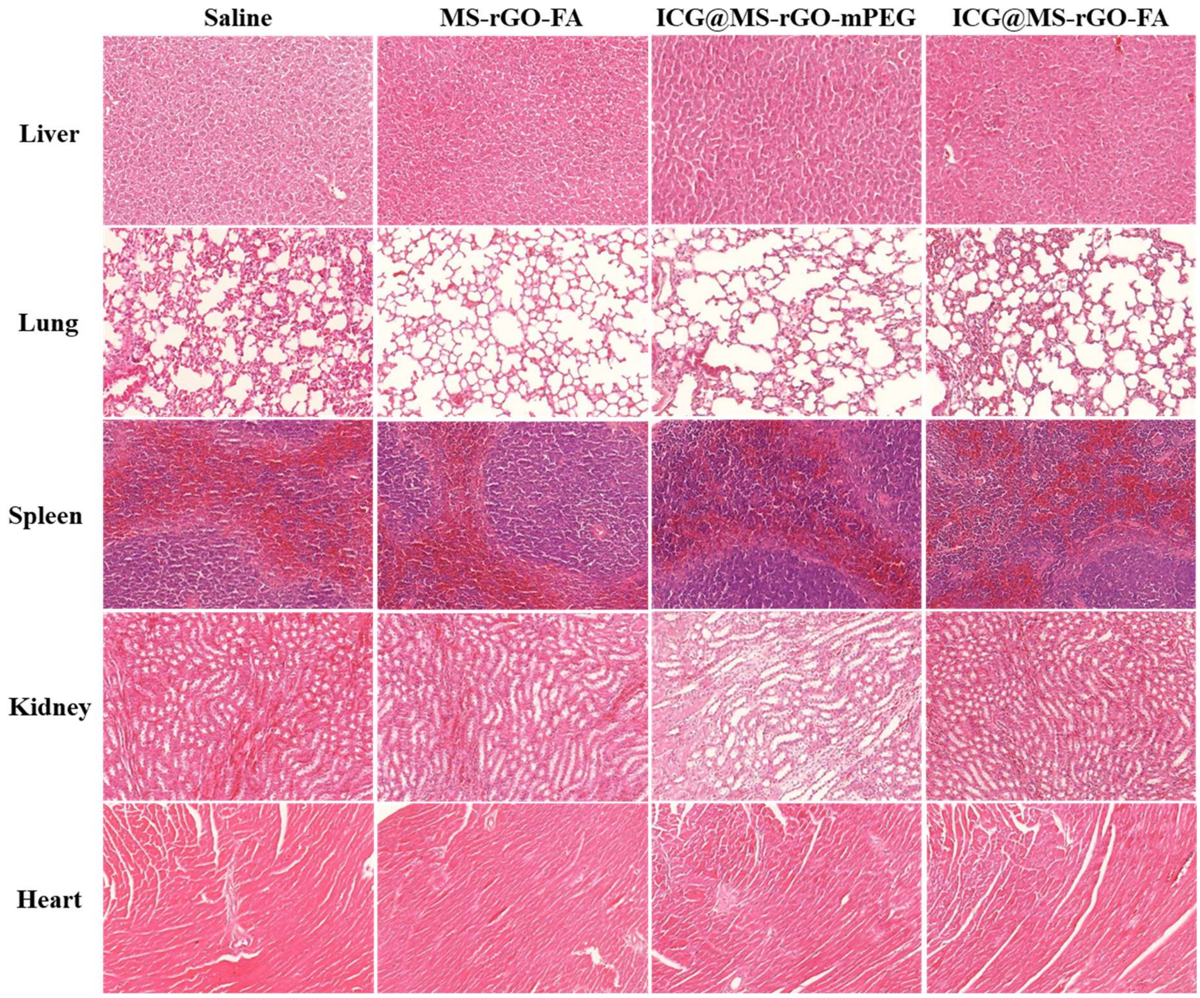

Figure S8. Histological images (H&E staining) obtained in major organs (e.g., liver, lung spleen, kidney, and heart) of the mice after treatment under various conditions.
